# Gestational Trophoblastic Neoplasia Treatment at the Butaro Cancer Center of Excellence in Rwanda

**DOI:** 10.1200/JGO.2015.002568

**Published:** 2016-04-13

**Authors:** Ignace Nzayisenga, Roanne Segal, Natalie Pritchett, Mary J. Xu, Paul H. Park, Edgie V. Mpanumusingo, Denis G. Umuhizi, Donald P. Goldstein, Ross S. Berkowitz, Vedaste Hategekimana, Clemence Muhayimana, Fidel Rubagumya, Temidayo Fadelu, Neo Tapela, Tharcisse Mpunga, Rahel G. Ghebre

**Affiliations:** **Ignace Nzayisenga**, **Natalie Pritchett**, **Paul H. Park**, **Temidayo Fadelu**, and **Neo Tapela**, Partners In Health–Inshuti Mu Buzima; **Vedaste E. Mpanumusingo**, **Denis G. Umuhizi**, **Vedaste Hategekimana**, **Clemence Muhayimana**, **Fidel Rubagumya**, and **Tharcisse Mpunga**, Rwandan Ministry of Health; **Rahel G. Ghebre**, Human Resources for Health Program Rwanda, Kigali, Rwanda; **Roanne Segal**, Ottawa University, Ottawa, Ontario, Canada; **Mary J. Xu**, **Paul H. Park**, **Donald P. Goldstein**, **Ross S. Berkowitz**, **Temidayo Fadelu**, and **Neo Tapela**, Harvard Medical School; **Paul H. Park**, **Donald P. Goldstein**, **Ross S. Berkowitz**, **Temidayo Fadelu**, and **Neo Tapela**, Brigham and Women’s Hospital, Boston, MA; and **Rahel G. Ghebre**, University of Minnesota Medical School, Minneapolis, MN, and Yale School of Medicine, New Haven, CT.

## Abstract

**Purpose:**

Gestational trophoblastic neoplasia (GTN) is a highly treatable disease, most often affecting young women of childbearing age. This study reviewed patients managed for GTN at the Butaro Cancer Center of Excellence (BCCOE) in Rwanda to determine initial program outcomes.

**Patients and Methods:**

A retrospective medical record review was performed for 35 patients with GTN assessed or treated between May 1, 2012, and November 30, 2014. Stage, risk score, and low or high GTN risk category were based on International Federation of Gynecology and Obstetrics staging and the WHO scoring system and determined by beta human chorionic gonadotropin level, chest x-ray, and ultrasound per protocol guidelines for resource-limited settings. Pathology reports and computed tomography scans were assessed when possible. Treatment was based on a predetermined protocol stratified by risk status.

**Results:**

Of the 35 patients (mean age, 32 years), 26 (74%) had high-risk and nine (26%) had low-risk disease. Nineteen patients (54%) had undergone dilation and curettage and 11 (31%) had undergone hysterectomy before evaluation at BCCOE. Pathology reports were available in 48% of the molar pregnancy surgical cases. Systemic chemotherapy was initiated in 30 of the initial 35 patients: 13 (43%) received single-agent oral methotrexate, 15 (50%) received EMACO (etoposide, methotrexate, dactinomycin, cyclophosphamide, and vincristine), and two (7%) received alternate regimens. Of the 13 patients initiating methotrexate, three had their treatment intensified to EMACO. Four patients experienced treatment delays because of medication stockouts. At a median follow-up of 7.8 months, the survival probability for low-risk patients was 1.00; for high-risk patients, it was 0.63.

**Conclusion:**

This experience demonstrates the feasibility of GTN treatment in rural, resource-limited settings. GTN is a curable disease and can be treated following the BCCOE model of cancer care.

## INTRODUCTION

Gestational trophoblastic neoplasia (GTN) involves clinical conditions including invasive moles, choriocarcinoma, placental site trophoblastic tumors, and epithelioid trophoblastic tumors. The establishment of referral cancer centers in high-income countries has led to centralization of GTN care, facilitating the conduct of clinical trials and development of expertise despite low patient volume in any one center.^1-4^ Collaborations and improvements in care have led to overall cure rates exceeding 90% in countries with a high level of resources for GTN treatment.^3,5^

However, challenges to GTN diagnosis and treatment exist in resource-limited settings. Approximately 50% of GTN cases arise from hydatidiform molar pregnancy, 25% from miscarriage or tubal pregnancy, and 25% from term pregnancy.^4,5^ Standard-of-care diagnosis of molar pregnancy is often dependent on the use of ultrasonography in the evaluation of pregnancy-associated bleeding and accurate testing for beta human chorionic gonadotropin (β-hCG), a tumor marker for GTN.^6-8^ Suction dilation and evacuation is an accepted procedure for the management and diagnosis of gestational trophoblastic disease, and accurate and timely pathology supports preliminary clinical diagnosis.^5,9^ In low-resource settings, manual vacuum suction is often practiced in the management of abortions. Access to ultrasonography in the rural setting can be limited, and pathology analysis is often not performed or available to facilitate early diagnosis of molar pregnancy. Furthermore, delays (both patient and system) often result in a longer time interval between the antecedent pregnancy and diagnosis of GTN, correlating with higher burden of disease, higher GTN risk scores, and possibly poorer outcomes.^10,11^

The Butaro Cancer Center of Excellence (BCCOE), located in the rural northern Burera district in Rwanda, is a pioneer in rural-based cancer treatment in low-resource settings.^12^ The center serves as a model of accessibility, where the cost of care is offset for the poor.^13^ GTN is a priority cancer because of its high cure rate, target population of healthy young women of reproductive age, and manageable subset of chemotherapeutic drugs. Treatment paradigms for GTN were adapted to available resources and designed for care delivery by nononcologists with remote clinical guidance by medical experts, given the limited national oncology capacity.^12^

This article aims to describe the management of GTN in resource-limited countries, report on treatment outcomes among patients referred to and treated at BCCOE in Rwanda, and identify factors associated with the potential for improved outcomes among patients with GTN. This is the first study to our knowledge to report on the GTN experience from a rural, resource-limited cancer center in Rwanda, paving the way for future adaptation of treatment models for this type of setting.

## PATIENTS AND METHODS

### Setting and Study Population

BCCOE was established in 2012 by the Rwandan Ministry of Health with support from its collaborators, the nonprofit organization Partners In Health (Boston, MA) and the Dana-Farber/Brigham and Women’s Cancer Center (Boston, MA). The center serves the Rwandan rural-based population in the Northern Province and has become a national referral center for select cancer care, evaluating 2,326 patients in its first 2 years.

A retrospective medical record review was performed for all patients assessed or treated for GTN between May 1, 2012, and November 30, 2014, at BCCOE in Rwanda. Thirty-five patients were identified for inclusion in this study. The study protocol was reviewed and approved by the Rwandan National Ethics Committee.

### Diagnosis and Treatment

Patients were treated for GTN on the basis of a protocol adapted to regionally available resources. Clinical suspicion combined with persistently elevated or rising β-hCG levels longer than 3 weeks after evacuation of a molar pregnancy prompted further work-up. On the basis of prespecified protocol guidelines, minimal diagnostic work-up included quantitative serum β-hCG level, physical examination, abdominal and pelvic ultrasound, and chest x-ray. Preferred evaluation when available included computed tomography (CT) scans of chest and abdomen; if positive, a CT scan of the head was thereafter included.

Stage, risk score, and risk category were based on International Federation of Gynecology and Obstetrics staging and modified WHO risk scoring system.^14,15^ Any laboratories or imaging reports not included in a patient’s medical record were assumed not to have been performed. Furthermore, given that protocols were adapted to resource availability, staging and risk scores were calculated on the basis of best available data. β-hCG values were obtained from three different laboratories in the country; units and reporting styles (exact result *v* range) varied among reports.

Before initiation of therapy, all patients were counseled on contraception. Chemotherapy was based on staging and risk classification. Patients with stage I or low-risk stage II or III disease (WHO risk score ≤ 6) were treated with single-agent methotrexate (MTX).^2,16,17^ In response to challenges in chemotherapeutic drug storage, patient compliance, and cost limitations, oral MTX was used as first-line treatment of low-risk GTN.^17^ Oral MTX was delivered at a dose of 0.4 mg/kg (maximum, 25 mg) once a day for 5 days every 2 weeks. Patients with stage IV or high-risk disease (WHO risk score ≥ 7) were received the combination chemotherapy regimen EMACO (etoposide, MTX, dactinomycin, cyclophosphamide, and vincristine).^3,4,18,19^ Quantitative β-hCG levels were recommended on day 1 of every cycle, and chemotherapy continued until normal β-hCG levels were reached. In line with principles of consolidation therapy in GTN management, patients received two additional cycles of chemotherapy after normalization of quantitative β-hCG values. Once therapy commenced, all β-hCG values were sent to the same laboratory.

Once a patient achieved a complete response, follow-up was recommended every month for 3 months and included complete clinical examination and quantitative β-hCG. Thereafter, surveillance examinations continued at an interval of every 3 months for a year. For patients whose β-hCG levels did not fall within the respective half-lives, persisted, or rose, a multidisciplinary call was organized with the appropriate surgical and medical consultants for individual and optimal care.

### Data Collection and Analysis

Data extraction was performed by two independent researchers (N.P., M.J.X.). Where questions or discrepancies existed, the opinion of a clinician was requested (I.N., R.S., R.G.G.). Medical record review was supplemented by the existing electronic medical records. Data were analyzed using STATA software (version 12; STATA, College Station, TX). The Kaplan-Meier plot was stratified by low- and high-risk GTN categories.

## RESULTS

### Patient Characteristics at Diagnosis

Patient characteristics at diagnosis are listed in Table 1. The average age of patients at diagnosis for low-risk disease was 32 years; for high-risk disease, it was 38 years. Patients had usually passed through at least one referral or district hospital before presenting to BCCOE. Four patients (11%) presented directly to BCCOE at initial diagnosis. All patients except one were confirmed to have negative HIV status at diagnosis. Data were unavailable on the remaining patient. Low-risk patients presented an average of 5 months from index pregnancy. High-risk patients had a longer average delay of 11 months from index pregnancy.

**Table 1:**
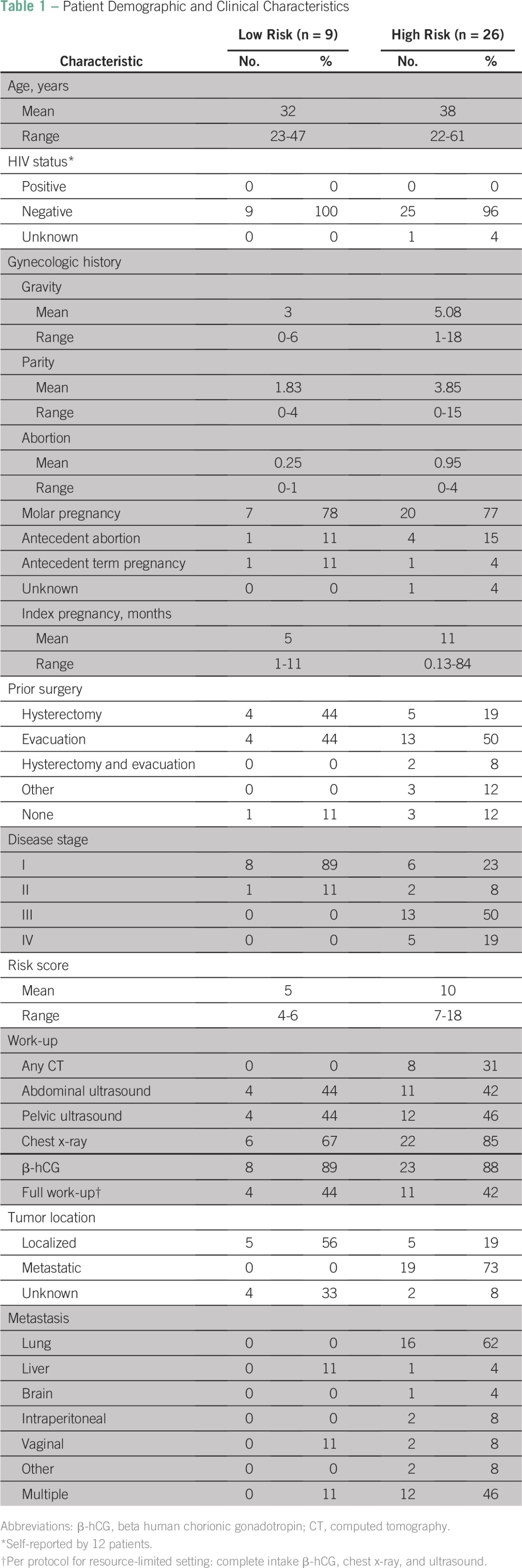
Patient Demographic and Clinical Characteristics

The majority of patients had an antecedent molar pregnancy (low risk, 78%; high risk, 77%). Patients also presented with an antecedent pregnancy with abortion or antecedent term pregnancy. Of the 35 patients, 19 (54%) had undergone evacuation and 11 (31%) hysterectomy. The three other prior surgical interventions performed included a cesarean section and two laparotomies. In total, 28 patients (low risk, 88%; high risk, 81%) had received some form of surgical evaluation related to gestational trophoblastic condition before arrival at BCCOE. Most patients (n = 29 [83%]) had not received any form of chemotherapy before presentation at BCCOE.

### Tumor Characteristics and Staging Work-Up

International Federation of Gynecology and Obstetrics staging and work-up results are listed in Table 1 (stage I disease, n = 14; stage II, n = 3; stage III, n = 13; stage IV, n = 5). The majority (74%) of patients were high risk, and nine (26%) were categorized as low risk. The distribution toward mostly high-risk patients with late-stage disease demonstrates the high level of acuity in patients presenting at BCCOE.

Less than half (43%; low risk, n = 4; high risk, n = 11) of patients received a full work-up as per protocol. The protocol for complete work-up at BCCOE includes intake β-hCG, chest x-ray, and liver ultrasound. Most patients (89%) had a β-hCG value available at intake, and 80% received a chest x-ray. Less than half (45%) of the patients received an abdominal or pelvic ultrasound, and only eight (23%) received any form of CT scan as part of their initial staging work-up.

With these data, 10 patients (29%) were determined to have a localized tumor, and 19 (54%) were found to have tumor metastasis. Among high-risk patients, disease had metastasized to the lungs in 16 (62%), to the liver in one (4%), to the brain in one (4%), intra-abdominally in two (8%), vaginally in two (8%), and to other sites in two (8%); 12 (46%) had two or more verified sites of disease.

### Pathology Reports

**Fig 1:**
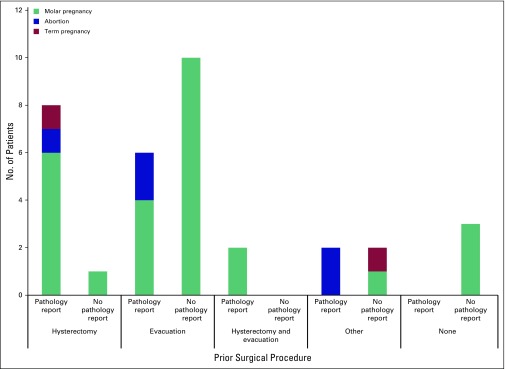
Gynecologic history and surgical history comparison.

The availability of pathology reports from any surgical interventions was reviewed. Of the 27 patients with antecedent molar pregnancy, seven had undergone hysterectomy, 14 had undergone evacuation, two had undergone both hysterectomy and evacuation, one had undergone laparotomy, and three had had no prior surgical intervention (Fig 1). Pathology reports were available from approximately half (48%) of the molar pregnancy surgical cases. Of the five antecedent abortions, one had undergone hysterectomy (pathology report available), two had undergone evacuation (no pathology available), and two had undergone laparotomy (no pathology available). Of the two patients with antecedent term pregnancy, one had undergone hysterectomy (pathology available), and one had had cesarean section (no pathology available). Between the two most common surgical procedures, hysterectomy was more likely to have pathology available (91% of cases) than evacuation (33% of cases).

### Treatment Course

Treatment course variables are listed in Table 2. First-line therapy for patients included the following: 13 patients received single-agent oral MTX, 15 received EMACO, and two received alternate regimens. Alternate regimens were provided because of medication stockouts; one patient with CNS metastasis received high-dose intravenous MTX, and the second patient with high-risk GTN received paclitaxel plus cisplatin and paclitaxel plus bleomycin doublet therapy. An additional three patients received EMACO as second-line therapy after MTX failure. For those patients receiving single-agent MTX, the average number of cycles was 5.3 (range, one to 18), with an average of 4.7 cycles (range, one to 12) required for normalization of β-hCG levels. For those patients receiving EMACO, the average number of cycles received was 4.6 (range, one to nine), with an average of 6.7 cycles (range, six to eight) required for normalization of β-hCG levels. The mean dose density for EMACO received by all patients averaging all cycles was 97% (range, 81% to 100%).

**Table 2:**
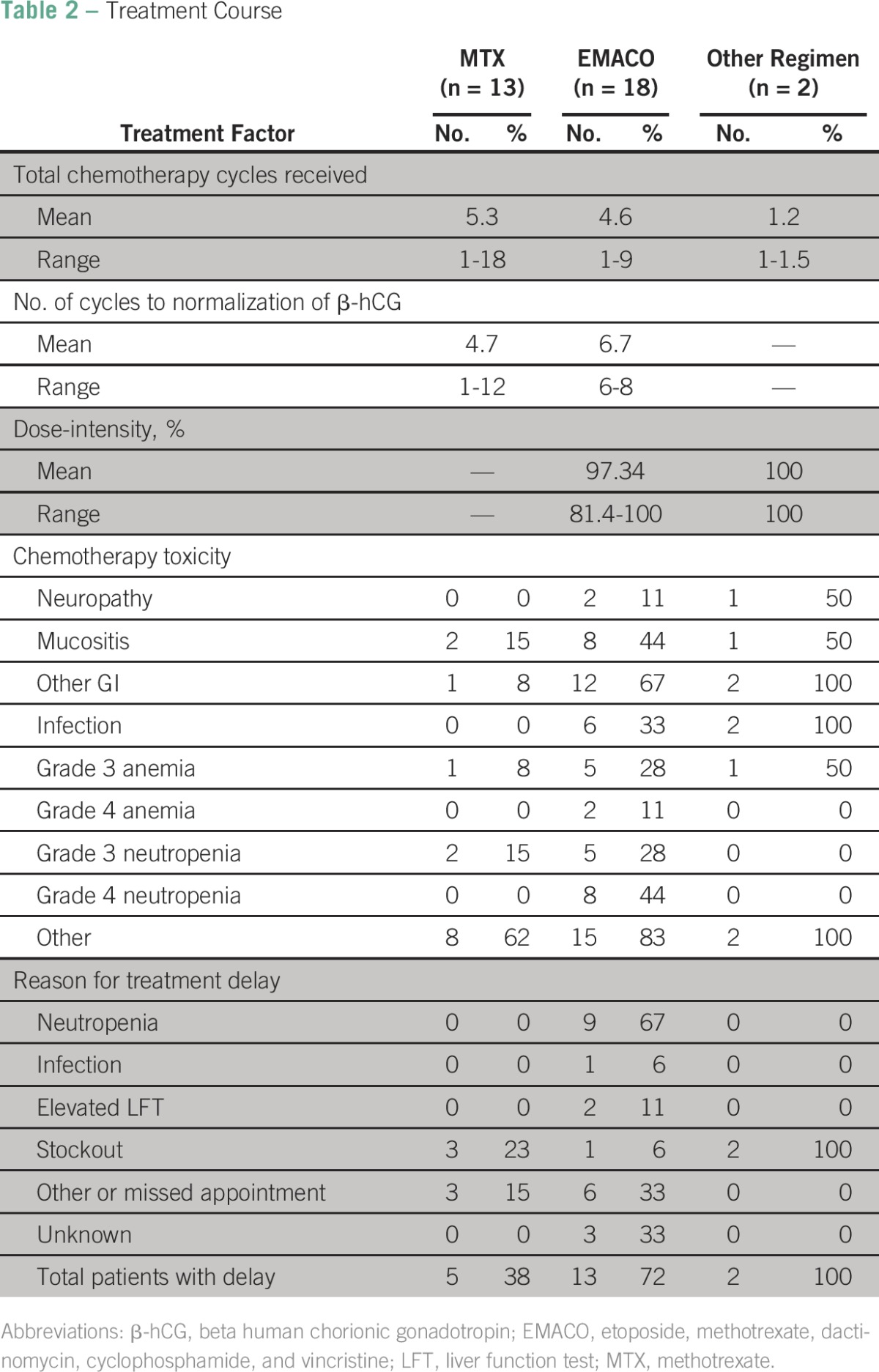
Treatment Course

### Toxicity

There were few reports of chemotherapy toxicity events with single-agent oral MTX (Table 2). Of the 14 events reported among patients receiving MTX, there were one grade 3 anemia and two grade 3 neutropenias. There was higher toxicity with the multidrug EMACO chemotherapy. There were 63 events reported with EMACO (Table 2). Hematologic toxicities included grade 3 to 4 anemia in seven cycles (39%) and neutropenia in 13 cycles (72%). Of the patients with neutropenia, six developed febrile neutropenia or other infections. Other adverse effects included generalized symptoms such as pain, loss of appetite, and weakness.

There were six treatment delays experienced by five patients receiving single-agent MTX. Three of the MTX delays resulted from medication nonavailability and three from missed appointments. Of the total 18 patients receiving EMACO, five did not experience any delays, whereas 13 experienced 38 documented delays. The causes for these treatment delays included neutropenia and associated complications on 22 occasions (average of two events per patient), infection not related to neutropenia in one patient, elevated liver function tests in two patients, one missed appointment each in six patients, medication stockout in one patient, and other reasons in six patients.

### Outcomes

**Fig 2:**
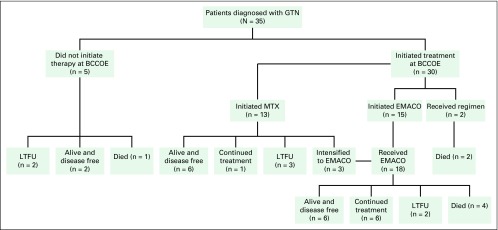
Treatment outcomes. BCCOE, Butaro Cancer Center of Excellence; EMACO, etoposide, methotrexate, dactinomycin, cyclophosphamide, and vincristine; GTN, gestational trophoblastic neoplasia; LTFU, lost to follow-up; MTX, methotrexate.

Of the 35 patients diagnosed at BCCOE, 30 initiated systemic chemotherapy. Five patients did not receive chemotherapy treatment at BCCOE; two of these were lost to follow-up after the initial consult, one died as a result of disease before therapy, and two were found to be disease free posthysterectomy for presumed invasive mole (Fig 2).

Outcomes for those patients treated (n = 30) are summarized in Table 3 and Figure 2. Of the 13 patients initiating single-agent MTX, six achieved normalized β-hCG levels, one continued to receive treatment, three were lost to follow-up, and three had their treatment intensified to EMACO. Of the 18 patients who received EMACO therapy, six achieved normalized β-hCG levels, six continued to receive therapy, four died as a result of disease, and two were lost to follow-up. Two patients received alternate regimens, one because of brain metastasis and one because of medication stockout; both died as a result of disease. A total of six patients died as a result of disease at an average follow-up time of 12 days (range, 5 days to 2 months).

**Table 3:**
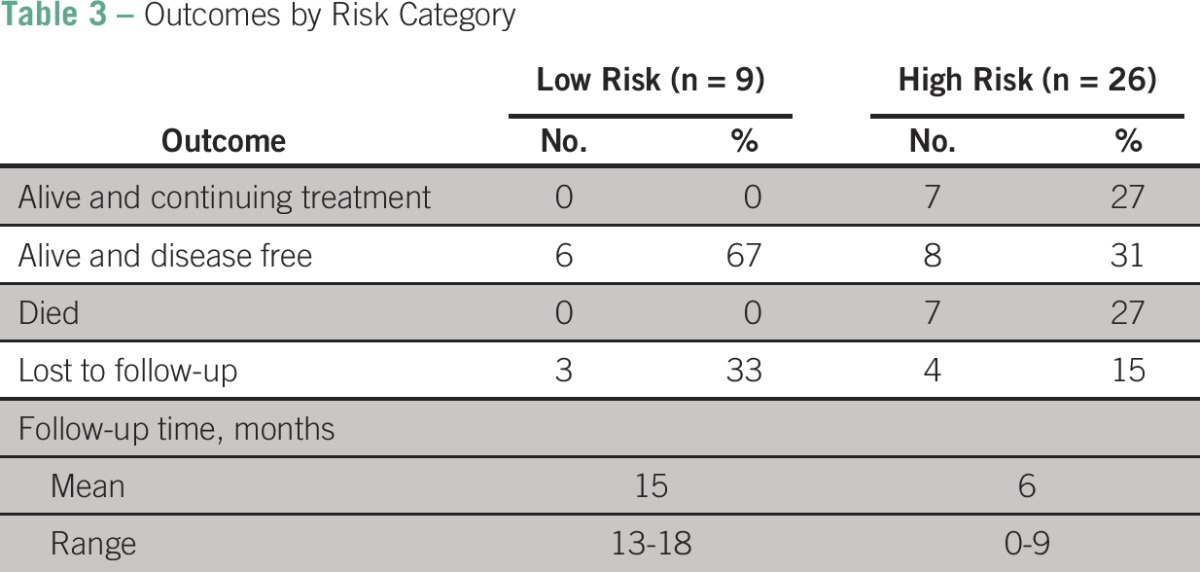
Outcomes by Risk Category

Overall, of the 30 patients receiving systemic therapy, 12 (41.4%) were alive and disease free, seven (24.1%) continued to receive therapy, six (20.7%) died, and five (13.8%) were lost to follow-up. For the remaining patients, the average length of follow-up time was 15 months for low-risk patients and 6 months for high-risk patients. When comparing risk category and outcome, the challenges of high-risk and late-stage presentation of patients become especially apparent. All patients who died while receiving treatment were high risk.

**Fig 3:**
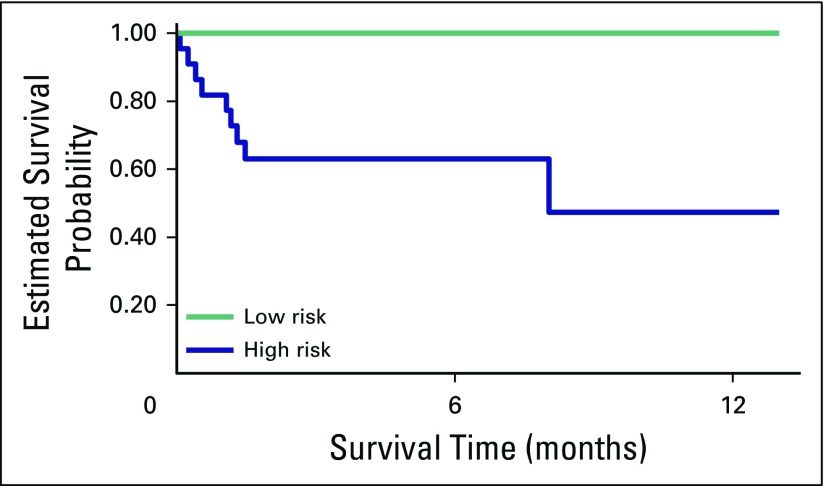
Kaplan-Meier survival curve by risk category.

Finally, a Kaplan-Meier survival curve that includes estimated 1-year survival probability from initiation of treatment at BCCOE is included in Figure 3. At a median follow-up of 7.8 months, the survival probability for low-risk patients was 1.00; for high-risk patients, it was 0.63.

## DISCUSSION

Cancer is one of the more challenging noncommunicable conditions to address in resource-limited settings because of its multidisciplinary nature, the high cost of care, and the need for skilled professionals. However, the BCCOE model serves as a step toward comprehensive cancer care provided by specialists. The protocol-driven treatment provided by nononcologists with guidance from specialists remotely has preliminarily led to successful treatment of GTN. Remote monitoring has included electronic consultation of individual patient cases and telemedicine oncology care conferences for complex cases. Despite many of the limitations in diagnosis and treatment of GTN, 100% survival for low-risk GTN and 63% for high-risk GTN, albeit with early follow-up data, in this study demonstrate the capacity of a rural low-resource oncology program to deliver much-needed cancer care.

Compared with those in other studies, survival outcomes for high-risk GTN at BCCOE fell below expected treatment outcomes in established GTN treatment centers.^20-22^ However, the Kaplan-Meier curves were highly influenced by the relatively short follow-up time. More-accurate comparisons may be made in the future with additional follow-up and an expanded patient population.

Successful treatment of patients at BCCOE has in part been based on foundational collaborations and an emphasis on the rural poor population the program serves.^12,23^ Led by the Rwandan Ministry of Health, care for all patients, including those with GTN, has been integrated into the existing health system, allowing for sustainable growth with early support from collaborators. Furthermore, given patient referral from across the country, communication among all national referral centers has been critical. Along with national and international collaborations, social service supports have led to higher patient retention rates than expected, given the socioeconomic landscape among our patients.

Although preliminary results are promising, a great limitation to the study is incomplete information, commonly encountered in clinical care and program evaluation in similar settings.^24,25^ As a result of limited resources, CT imaging was not mandated in the initial protocol. Consequently, only 30% of high-risk patients received chest and abdominal CTs, likely leading to understaging in many patients. Patients with MTX resistance received EMACO as salvage chemotherapy. Single-agent dactinomycin can be used as second-line chemotherapy, but because of concern surrounding understaging of patients and risk of loss to follow-up, escalation to EMACO in lieu of single-agent dactinomycin was incorporated into the GTN BCCOE treatment protocol.^2,4,5^ Even when health services were available, as among patients who were surgically treated for antecedent molar pregnancy, only 52% of patients had pathology reports available. Both incomplete information and incomplete documentation affect appropriate risk stratification, a challenge in GTN management that has been noted in cancer centers in low-resource settings.^21,26^ Additional study limitations include the short duration of follow-up and information only on patients with GTN at BCCOE, providing a partial representation of the country.

Moving forward, continuing to bolster health infrastructure will improve care of patients with GTN. At diagnosis, additional imaging for metastatic work-up will more accurately stage disease and guide appropriate therapy. Specific to GTN, available and timely β-hCG levels direct patients’ clinical course. Ideally, levels would be available at the start of each new chemotherapy cycle; however, only a few laboratories in the country evaluate β-hCG levels, many using differing measurement scales, leading to confusion in clinical care and evaluation. Increased or decentralized access to β-hCG testing along with standardized measurement could lead to earlier diagnoses and more-accurate treatment monitoring.

Programs treating GTN should also ensure supply of necessary chemotherapeutic agents and consider supplemental medications that support treatment response. Relatively consistent access to critical chemotherapy agents was achieved, with an average of 4.7 cycles of MTX and 6.7 cycles of EMACO provided to patients to achieve complete response.

Nevertheless, two patients’ chemotherapy was delayed by stockouts of EMACO agents, one of whom then received an alternate regimen and later died. Although a majority of deaths were attributed to disease, EMACO is a myelosuppressive regimen that led to grade 3 or 4 neutropenia in 72% (13 of 18) of patients thus treated. Clinical guidelines include the use of granulocyte colony-stimulating factor to reduce risk of treatment delays resulting from neutropenia; however, because of cost, these agents were not available to our patients.^9^ Although still costly despite recently becoming available in generic formulary, granulocyte colony-stimulating factor could be considered for neutropenic patients in subsequent cycles.

The current protocol is tailored to resource-limited settings; however, nuanced modifications could enhance care of high-risk patients with GTN. Using the current protocol, all deaths in the GTN cohort occurred early in the course of treatment, reflecting high disease burden. Other studies have modified chemotherapy regimens for patients with high disease burden to reduce early death (< 4 weeks after initiation of treatment).^22,27^ Such treatment modifications in select patients could improve overall outcomes.

Longer-term studies can present a more accurate portrait of patient outcomes and barriers to care in GTN in rural resource-limited settings. Both at BCCOE and at decentralized health facilities, understanding of barriers to care and disease awareness among care providers will help identify areas to enhance care delivery. Need for cancer care is anticipated to grow in Rwanda, and data that allow for scaling up of cancer care that is both effective and cost efficient are lacking. In addition, system-based barriers, including lack of pathology analysis at the local health facility, must be addressed with innovative solutions. Finally, although social support has been beneficial to patients both anecdotally and in other studies, more careful analyses of benefits and gaps in services may further increase patient retention. These lessons will help optimize rural-based cancer models for GTN.

In conclusion, our study supports GTN as a priority disease, given that patients tend to be young, the disease is highly curable, and few medications with manageable toxicities are required. The BCCOE model involving protocol-based cancer care by mentored nononcologists has successfully increased access to GTN treatment in rural settings. Preliminary outcomes support the importance of integrated care by oncologists and local nononcologists, prioritizing diagnostic and management tools, protocol modifications, integrated monitoring and evaluation, and social support in resource-limited settings. Optimal models for resource allocation in cancer care can best be developed in the context of resource-limited health systems similar to BCCOE yet applicable in all settings.
